# Distinct groups of repetitive families preserved in mammals correspond to different periods of regulatory innovations in vertebrates

**DOI:** 10.1186/1745-6150-7-36

**Published:** 2012-10-25

**Authors:** Jerzy Jurka, Weidong Bao, Kenji K Kojima, Oleksiy Kohany, Matthew G Yurka

**Affiliations:** 1 Genetic Information Research Institute, 1925 Landings Drive, Mountain View, CA, 94043, USA

**Keywords:** Transposable elements, Conserved repeats, Genetic drift, Evolution

## Abstract

**Background:**

Mammalian genomes are repositories of repetitive DNA sequences derived from transposable elements (TEs). Typically, TEs generate multiple, mostly inactive copies of themselves, commonly known as repetitive families or families of repeats. Recently, we proposed that families of TEs originate in small populations by genetic drift and that the origin of small subpopulations from larger populations can be fueled by biological innovations.

**Results:**

We report three distinct groups of repetitive families preserved in the human genome that expanded and declined during the three previously described periods of regulatory innovations in vertebrate genomes. The first group originated prior to the evolutionary separation of the mammalian and bird lineages and the second one during subsequent diversification of the mammalian lineages prior to the origin of eutherian lineages. The third group of families is primate-specific.

**Conclusions:**

The observed correlation implies a relationship between regulatory innovations and the origin of repetitive families. Consistent with our previous hypothesis, it is proposed that regulatory innovations fueled the origin of new subpopulations in which new repetitive families became fixed by genetic drift.

**Reviewers:**

Eugene Koonin, I. King Jordan, Jürgen Brosius.

## Background

Transposable elements (TEs) are DNA segments that are capable of reproducing and inserting themselves into genomic DNA using a number of different mechanisms. Typically, some TEs remain active for a limited period of time during which they produce mostly inactive copies of themselves known as families of interspersed repetitive elements, or repeats. The TE families are preserved in eukaryotic genomes over an extended period of time but the number of elements per family declines with time due to attrition of non-functional DNA, also referred to as “junk DNA”. Some of the TE-derived repeats or repeat fragments originally fixed in the population may become recruited as functional components of the non-coding genomic DNA [1-8], and those can be identified in DNA segments conserved across species [9,10]. Thus, the overall number of repetitive elements in the conserved genomic regions, relative to the total number of repeats in the entire genome, is expected to grow over time, primarily due to attrition of the repetitive DNA that didn’t assume any functional role in the genome. We use this process to identify ancient repetitive families of different age.

## Results and discussion

### Analysis of ancient families of TEs

We selected 381 consensus sequences from Repbase [11], which represent repetitive families present in all mammalian species sequenced to date. We also report an additional 152 families of conserved repetitive elements (Additional file 1: Table S1, column F; consensus sequences to be released in Repbase), which were identified and reconstructed using the human genome sequence data (see Methods). The total set of 533 consensus sequences present in all mammals (listed in Additional file 1: Table S1, column C), was screened against the human genome, using Censor [12], to determine the genomic count of repetitive elements for each family. The same set was screened separately against the previously published human genomic regions conserved among different vertebrates including mammals and birds, and representing ~5% of the human genome [10]. Using binomial and chi square statistics we identified 266 families that are significantly overrepresented in the conserved regions relative to the rest of the human genome, which are hereafter referred to as families of “conserved repeats”. Most of the 266 consensus sequences, with the median length of ~238 bp, are likely fragments of ancient repetitive elements. To date, ~44% of them were classified as belonging to a particular group of transposable elements based on the presence of TE-specific features or similarity to known TEs.

**Figure 1:**
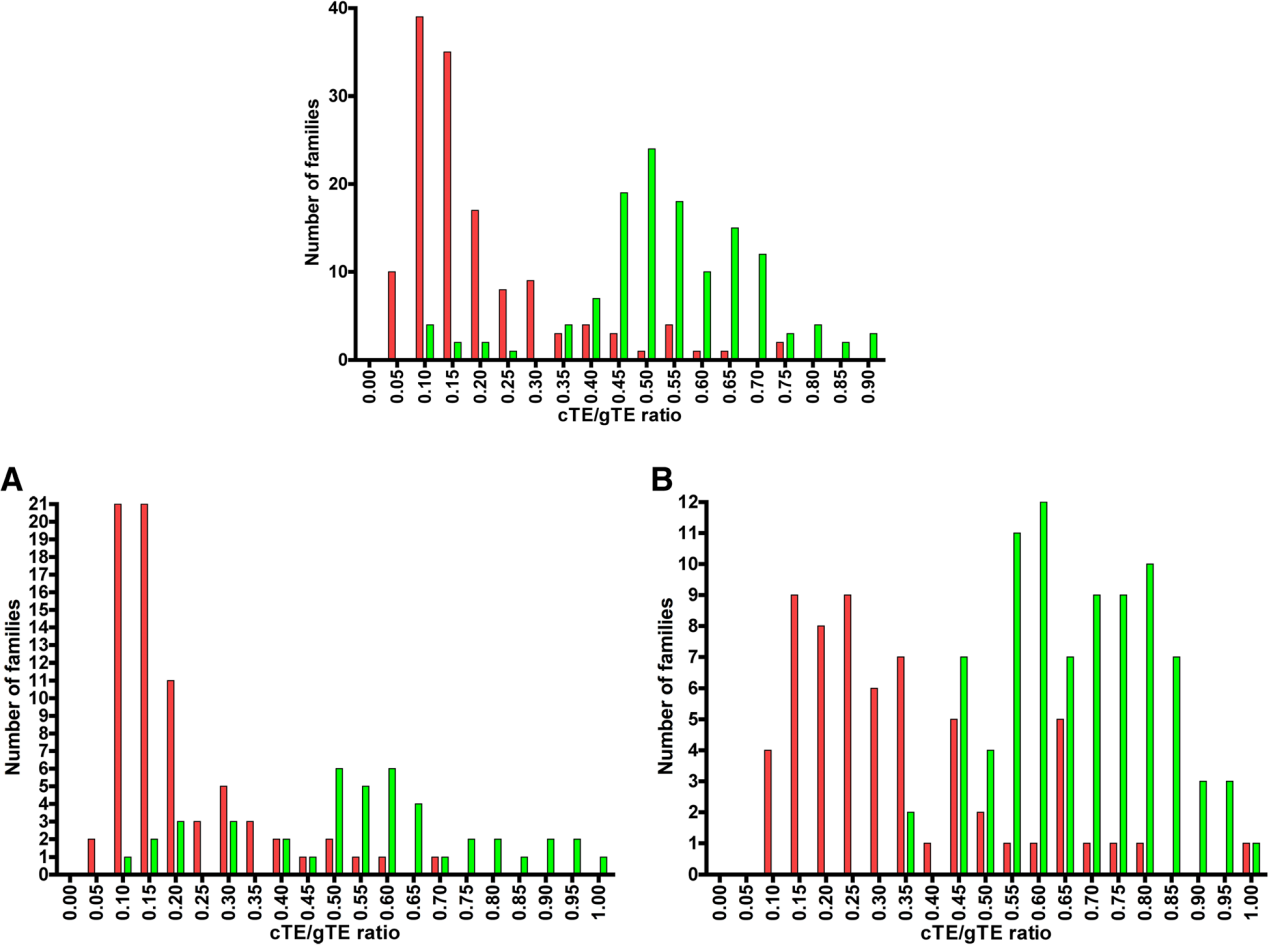
**Frequency distribution of 266 conserved repetitive families based on their repeat counts in the conserved regions (cTE) and in the entire human genome (gTE).** Each family is counted in the interval (bin) with its corresponding cTE/gTE ratio (vertical axis). The red bars indicate families present in mammals only, and the green bars represent families shared by mammals and chicken. **A.** Frequency distribution of 119 classified families of conserved TEs, analogous to that in Figure 1. **B.** Frequency distribution of 147 unclassified families of conserved TEs, analogous to that in Figure 1.

For each of the 266 families, we divided their numbers of repeats present in the conserved regions (cTE) by their total numbers in the human genome (gTE). Figure 1 presents the cumulative numbers of families corresponding to different cTE/gTE ratios for the entire set of 266 families. The families cluster around two major peaks separated by a minimum around 0.35. The first peak corresponds mostly to younger repetitive families present in mammals only (Figure 1, red), and the second one represents more ancient families shared by mammals and chicken (Figure 1, green). The clustering is similar for families positively identified as derived from TEs (Figure 1A) and for yet unclassified repetitive families (Figure 1B). Thus, the cTE/gTE ratios can be used to approximately rank the conserved repetitive families from the youngest to the oldest ones.

**Figure 2:**
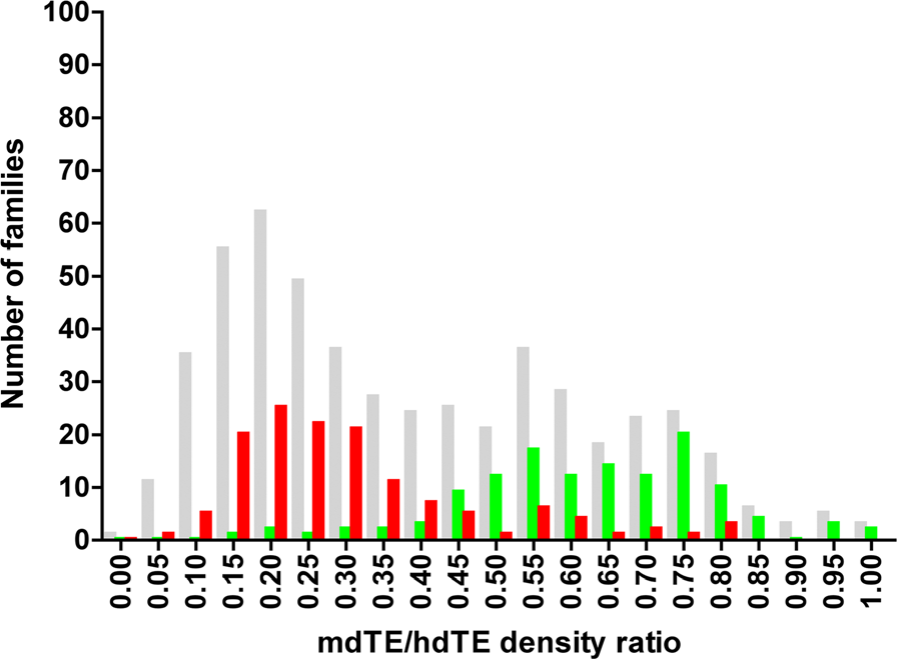
**Frequency distribution of the mammalian-wide families based on the ratio of repeat densities in the mouse and in the human genome.** The repeat densities for each family were calculated per 1 Mb of the mouse (mdTE) and of the human genome (hdTE). Each bar represents the number of families (vertical axis) with their mdTE/hdTE values within the same interval (bin). The grey bars illustrate the distribution of all mammalian-wide families except of those with mdTE/hdTE > 1 (for details see Materials and Methods). The red bars represent a subset of conserved families present in mammals only, and the green bars represent a subset of families shared by mammals and chicken.

A comparable clustering can be obtained using a different approach based on the ratio of repeat densities per 1 Mb of the mouse (mdTE) and of the human genome (hdTE). The ratio reflects a faster attrition of non-essential DNA in mouse than in human, driven by differences in the mutation rates between the two [13]. In Figure 2, the 266 families of conserved repeats are also separated into a younger group present in mammals only (red bars), and the older one shared by mammals and chicken (green bars), based on the mdTE/hdTE ratio. The grey bars represent most of the 533 mammalian-wide families (for details see Materials and Methods). The two clusters of conserved families presented in Figure 1 and Figure 2, are likely to be remnants of at least two major outbursts of repeat amplification – one predating the separation of the mammalian and bird lineages and the other one corresponding to subsequent diversification of the ancient mammals prior to the origin of eutherians. 

**Figure 3:**
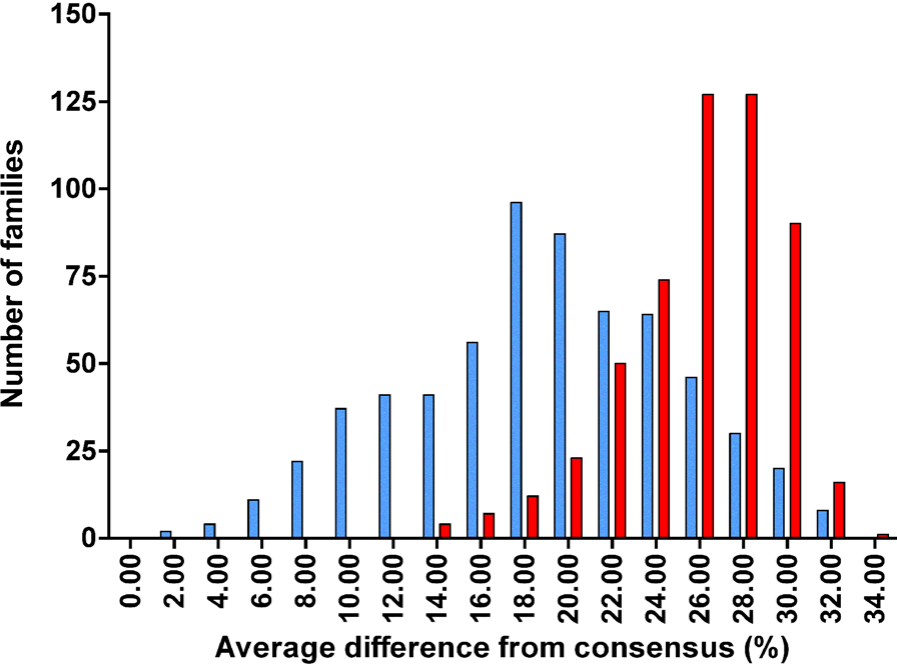
**Numbers of the human-specific families (blue bars) and mammalian-wide families of TEs (red bars), corresponding to average sequence differences of individual repeats from their respective family consensus sequences.** Average sequence difference equals 100 minus the average percentage of sequence identity to the family consensus sequence.

**Figure 4:**
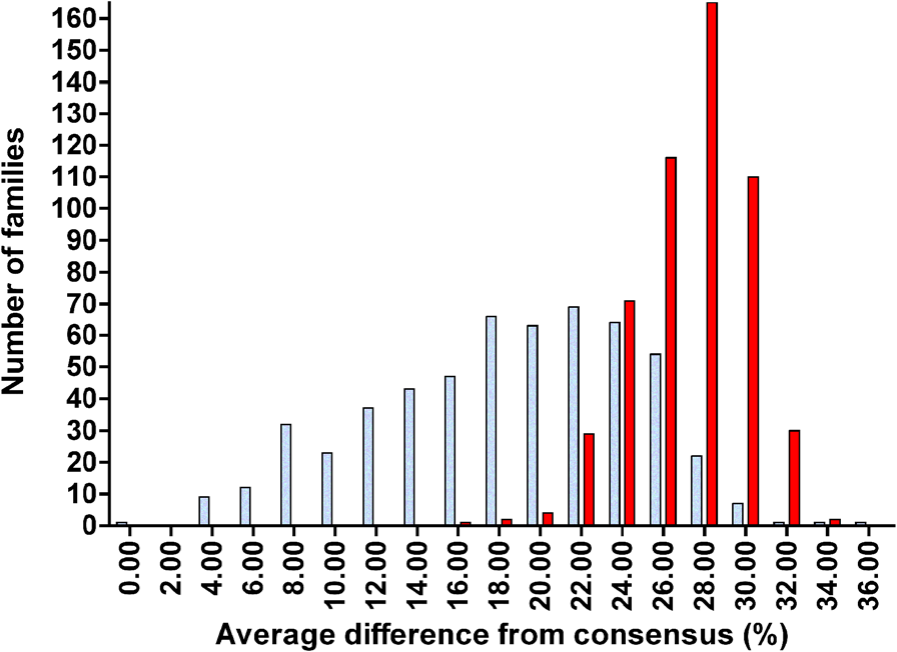
**Numbers of the mouse-specific families (blue bars) and of the mammalian-wide families of TEs (red bars), corresponding to average sequence differences of individual repeats from their respective family consensus sequences.** Average sequence difference equals 100 minus the average percentage of sequence identity to the family consensus sequence.

Clustering of repetitive families is not only found among the ancient families of conserved repeats, but also among mammalian repeats that are species and lineage-specific. In Figure 3 a separate, younger cluster (blue bars) is represented by the “human-specific” families, partially overlapping with the cluster of the entire set of 533 mammalian-wide families (red bars), based on sequence differences between individual repeats and their family consensus sequences (horizontal axis). The "human-specific" families of TEs are defined as those present in humans and in primates ancestral to humans (listed in Additional file 1: Table S1, col. A). In Figure 3, the mammalian-wide families form a single distribution rather than the bimodal distribution presented in Figures 1 and 2 because the separation of the more ancient families of TEs cannot be accomplished simply based on their sequence identity to consensus. Noticeably, the decline in the number of mammalian-wide families in Figure 3 coincides with the outburst of the human-specific families. A strikingly similar distribution pattern can be seen for a different set of TEs from the mouse genome (Figure 4, blue bars). The number of mouse/rodent-specific families also began growing rapidly during the decline in the number of mammalian-wide families (Figure 4, red bars).

Remarkably, the clusters shown in Figures 1,4 also largely correspond to independently identified periods of regulatory innovations [14]. The most ancient period, prior to the separation of mammalian and bird lineages, was dominated by regulatory innovations near transcription factors controlling the key developmental (trans-dev) genes [14]. The second period, approximately between 300 and 100 million years ago (Ma), was characterized by a gradual decline in changes near trans-dev genes accompanied by a high frequency of regulatory innovations near receptors of extracellular signals. A possible third period of regulatory innovations can be also seen in placental mammals [14].

### Repetitive families, population structure and speciation

Outbreaks of new TE families are often lineage and species-specific [15-24], which suggests a potential involvement of TEs in speciation. Such an involvement has been repeatedly explored since the eighties but no general explanation has emerged that would address the observed correlation between the origin of repetitive families and speciation (reviewed in [24,25]). The subject is attracting a renewed attention thanks to the growing avalanche of the genomic data on repetitive families. A recently published TE-Thrust hypothesis [26] correlates a massive activity of TEs with speciation, which indirectly links the origin of repetitive families and the origin of species. Specifically it states that:

"“…there is differential survival of those lineages that contain or can acquire suitable germline repertoires of TEs, as these lineages can more readily adapt to environmental or ecological changes, and can potentially undergo, mostly intermittently, fecund radiations.” "

The critical applicable questions are: (1) what is the suitable repertoire of TEs, and (2) why the entire sets of TEs expand into multiple families only at certain times in the history of a lineage and then decline? Our data indicate that major expansions of repetitive families were triggered and inactivated at least several times during the history of the mammalian lineage. In the original version of their hypothesis, the authors attribute the explosion of new repetitive families to “waves of TE infestation” [27]. Even if a substantial acquisition of active TEs by a population were taking place, there is no compelling evidence that their activities alone would automatically lead to a massive fixation of new families and new adaptations.

Our alternative hypothesis, also referred to as the carrier subpopulation (CASP) hypothesis [24], emphasizes the fundamental role of the population structure as the key factor driving the expansion of repetitive families and their impact on genome evolution. It specifically states that most populations carry random repertoires of active TEs, which can have mutagenic impact due to fixation of multiple copies in the population [24]. It further states that small populations are the primary incubators of repetitive families, due to fixation of TEs by genetic drift. Therefore, the fixation of TEs depends strongly on factors affecting the population structure of the host. The factors include major biological breakthroughs or cumulative innovations that can lead to new adaptations, or environmental changes that can open new niches due to extinctions of competitors. Such factors are likely to promote the increase in the number of small sub-populations exploring and adapting to new niches. The hypothesis implies that TEs are mere multiplying opportunists that may contribute to evolutionary changes mostly due to their mutagenic activities in small sub-populations. Interestingly, their highest mutational impact coincides with explorations of new adaptations by small subpopulations.

TE-generated mutations are essentially stochastic in nature, and many of them will have negative impact on survival. Small populations are much more vulnerable to extinction than the large ones [28], but those which survive are likely to become repositories of multiple pre-screened mutations that can be carried back to the meta-population or exchanged between different subpopulations by crossbreeding. Thus, multiple extinctions of small sub-populations may be an important evolutionary path of weeding out harmful mutations. Some of the surviving subpopulations may continue to diverge due to accumulation of TEs until they become founder populations for new species. A successful crossbreeding between individuals from diverse subpopulations may also lead to an abrupt emergence of new species [29].

### Biological innovations and the diversity of repetitive families

The results presented in this paper illustrate a recurrent rise and fall in the number of new TE-derived repetitive families during the history of the mammalian lineage, which coincides with evolutionary periods when different types of regulatory innovations began and ended. According to the CASP hypothesis [24], the rise and fall in the number of the repetitive families was caused by the rise and fall in the number of viable subpopulations that originated during that period. The origin of new subpopulations was probably fueled by adaptive advantages introduced by changes in gene regulation. Population subdivisions facilitate the fixation of TE insertions by genetic drift. The fixed TEs may become the raw material for biological innovations. The recruitment or elimination/attrition of inserted TEs is on a timescale that far exceeds the lifespan of any subpopulation. Moreover, regulatory innovations do not necessarily coincide with TE fixation. Once a regulatory innovation takes place, the population is likely to explore new niches, and to become subdivided again.

Based on this scenario, the mammalian genomic DNA combines families of TEs incubated in countless subpopulations over hundreds of millions of years. Most of the TE copies deposited in the genome are presumed neutral, and they are lost over time by attrition. A minority of TEs became exapted and survived as a conserved DNA. The attrition of TEs continues at a relatively steady rate as indicated by the time-dependent increase in the fraction of conserved TEs, relative to the total number in the genome.

It remains to be elucidated why a particular pattern of regulatory changes continued for hundreds of millions of years. Overall, the data imply that each pattern of regulatory innovations yielded diminishing adaptive advantages over time and eventually became exhausted. The cycle was repeated again as soon as new productive types of regulatory innovations came into play.

In summary, the observed outbursts of ancient families of TEs might have been associated with the origin of small subpopulations exploring the regulatory innovations in different ecological contexts. Crossbreeding between individuals from different subpopulations increased the number of different TE families in the surviving populations. The populations likely went through multiple subdivisions and crossbreeding during the exploration of regulatory innovations. After each extended period of innovations ran its course, the number of viable subpopulations declined and fixation of new repetitive families declined with it. The process was resumed when new types of regulatory innovations came into play during subsequent evolutionary periods.

## Conclusions

Ancient families of conserved repeats in mammalian genomes belong to distinct groups of repetitive families. The origin of different groups of families coincides with different periods of regulatory innovations [14]. Based on a recent hypothesis describing the origin of repetitive families [24], it is proposed that regulatory innovations increased the number of small viable subpopulations of individuals with new biological adaptations. New families of TEs became fixed in these subpopulations by genetic drift and preserved in the lineage leading to modern mammals.

## Methods

### Analysis of repetitive families in the human genome

Using Censor [12], we masked the entire human genome sequence [30] (version18, March 2006) by running it against the library of the human and ancestral mammalian repeats extracted from Repbase [11]. We also used Censor to identify new repetitive sequences in the human genome by systematically screening different sequence portions of the masked genome against each other. The multiple copies of the newly identified sequences were clustered into families based on sequence similarity and used to generate consensus sequences. All newly generated consensus sequences were screened again against the entire masked human genome and the next generation of consensus sequences was obtained from the new sets of identified repeats with incrementally expanded flanking regions. The process was repeated until screening of consensus sequences against the genome produced consensus sequences of stable lengths. All consensus sequences were analyzed manually for presence of characteristic hallmarks such as terminal inverted repeats (TIRs), open reading frames and similarity to known families of repeats. The annotated sequences of the newly identified 152 families will be released in Repbase upon publication of the article.

The newly identified and refined consensus sequences were combined with the remaining human and ancestral mammalian-wide consensus sequences from Repbase, and the entire set was screened against the original (unmasked) version of the human genome, using both Censor and RepeatMasker [31], to determine the number of repeats for each family. The same set was screened against the previously published conserved fraction of the human genome [10].

### Statistical evaluation

The entire output was analyzed statistically to determine families with repeat numbers significantly overrepresented in the conserved fractions representing ~4.8% of the human genome, relative to the remaining portion of the genome (95.2%). We compared the observed repeat count in the conserved and non-conserved regions to expected repeat count based on uniform distribution. We identified 266 families with repeat numbers significantly higher than expected in the conserved regions (P<0.05, see Additional file 2: Table S2). An additional 267 families present in all mammals are not significantly overrepresented in the conserved portion of the human genome used in this analysis.

### Comparison of repeat densities in mouse and human

All 533 mammalian-wide repeats (Additional file 1: Table S1, column C), and mouse-specific sequences from Repbase (listed in Additional file 1: Table S1, column B), were combined. The entire set was screened against the mouse genome [32] (version 9, July 2007), using Censor, to determine the number of repeats for each mammalian-wide family. We calculated the number of TE elements from each family per 1 Mb of the mouse genome (mdTE). Analogous densities were determined for the mammalian-wide repeats present in the human genome (hdTE). In general, the mdTE/hdTE ratio is expected to range from 0 to 1 due to a faster elimination rate of non-essential DNA in mouse than in human, driven by a higher mutation rate in rodents than in human [13]. However, some of the mammalian-wide families might have continued to expand in the rodent lineage but not in the primate lineage after the two separated, which would bias the mdTE/hdTE ratios towards higher values. Of the 533 mammalian-wide families, 26 were eliminated based on mdTE/hdTE ratio >1. The remaining ones were used in analysis presented in Figure 2.

## Abbreviations

TE(s): Transposable element(s); TIR(s): Terminal inverted repeats; Mb: Megabase (1,000,000 base pairs).

## Competing interests

None.

## Authors’ contributions

JJ designed the study and wrote the manuscript. All authors contributed to data analysis. WB and KKK contributed both to data analysis and revisions of the manuscript. All authors read and approved the final manuscript.

## Reviewers’ comments

Reviewer 1: Eugene V. Koonin, NCBI, NLM, NIH, Bethesda, MD 20894, USA

This is a very straightforward paper that links the fixation of different families of TEs in mammalian genome with major evolutionary transitions (here described as periods of regulatory innovations). I think the following conclusion that closes the article “it is proposed that regulatory innovations increased the number of small viable subpopulations of individuals with new biological adaptations. New families of TEs became fixed in these subpopulations by genetic drift and preserved in lineages leading to modern mammals” is quite correct. The proposed chain of causation here is: regulatory innovations -> subdivision of the ancestral populations into small subpopulations -> fixation of propagating TEs via drift. I wonder if an alternative interpretation could be at least as plausible: population bottleneck -> regulatory innovations+fixation of TE via drift? Regardless, the title of the article and some language, especially in the abstract, seem to imply direct contribution of TEs to the co-temporal regulatory innovations. However, one should avoid falling prey to ‘post hoc ergo propter hoc’: both regulatory innovations and bursts of TEs could be triggered by the same population-genetic factors, and these need not be adaptive.

**Author's response:***Indeed, regulatory innovations either due to fixation of TEs or other mutations can occur in small (lucky) populations. We elaborated on this point in the revised version “Biological innovations and the diversity of repetitive families”.There is also a broader point inspired by this and the last review that deserves closer attention. Specifically, small subpopulations are more likely to go extinct due to harmful mutations than to benefit from advantageous mutations. Nevertheless, the subpopulations that survive can become the treasure troves of pre-screened mutations (beneficial, neutral and slightly harmful), which can be transferred back to the meta-population by crossbreeding. Therefore, the existence of multiple co-temporal sub-populations may be critical for successful weeding out harmful mutations and passing the remaining ones for further testing by natural selection (see “Repetitive families, population structure and speciation” in the main text).*

Reviewer 2: King Jordan, Georgia Institute of Technology, USA

Jurka and colleagues present an analysis of repeat sequences, many of which have been deeply conserved across mammalian evolution. Detailed analysis of such sequences allowed the authors to identify distinct clusters of such repeats, three groups of which show marked expansions/contractions corresponding to previously characterized periods of regulatory innovation. Similar observations have been reported previously and have been taken to suggest that repeat families, transposable element (TE)-derived sequences in particular, may have provided raw material (e.g. novel regulatory sequences) that facilitated regulatory innovations during such periods of evolutionary transition (Oliver and Greene 2009 Bioessays 31:703; Oliver and Greene 2011 Mobile DNA 2: 8). Interestingly, the authors of this work favor a very different explanation for the expansion of repeats at such times. They hold that these periods of regulatory innovation were likely to be characterized by elevated levels of population subdivision, which in turn allowed for the fixation of numerous TE families by genetic drift. In this sense, the abundance of TE families can be considered to be a by-product of the population dynamics of the species whose genomes bear the elements rather than a driving force of evolutionary innovation (Jurka et al. Biol Direct 2011 6:44). This manuscript is therefore in some sense a continuation of an ongoing debate in the literature regarding the global role of TE family expansions and contractions in regulatory innovation, speciation and evolution. The new data the authors bring to bear on the question is a welcome addition to the debate. One thing that is missing from the discussion of the results, however, is a consideration of the evidence in favor of their very different world view compared to the groups who favor a more active role for TEs in driving regulatory innovation and evolution. Below, I pose a series of questions regarding the findings and how they relate to this debate.

As an aside it is worth noting that this paper reports a major addition of consensus sequences for newly identified human and mammalian conserved repeats, all of which have been deposited in Repbase. These consensus sequences represent and important resource for the research community and a new source of information and annotations for the human genome.

Major Points 1. The work of Oliver and Greene on the genomic drive or TE-thrust hypothesis for the role of TEs and regulatory innovation, which has direct relevance to this manuscript as discussed above, is not cited or discussed. Given that this work and the TE-thrust hypothesis articulate two clearly distinct perspectives on the role of TEs, in particular whether they are active agents or passive by standers, in the processes of regulatory innovation, speciation and evolution, these papers should be cited and discussed in the context of the current work (Oliver and Greene 2009 Bioessays 31:703; Oliver and Greene 2011 Mobile DNA 2: 8). For instance, if the authors disagree with the previous perspectives on an active role for TEs in driving regulatory innovation and speciation, they should state why. Or perhaps, if they feel these two world views are not actually mutually exclusive this could be discussed. But to ignore the works reporting a conflicting perspective is a mistake in my view.

**Author’s response:***In the original version of the manuscript we mentioned speciation only in passing as it was discussed in our hypothesis published a year earlier. Clearly, the issue continues to be of substantial interest because it was brought up in this and the last review. We appreciate the comments, and we elaborated on this point in the revised version (see “Repetitive families, population structure and speciation”). TEs have an evolutionary impact due to their mutagenic activities, but they are not the “drivers” of evolution. The fixation of any mutations, including the TE-generated ones, depends to a large extent on the population structure, which in turn is driven by biological innovations and ecological factors. The original “genomic drive hypothesis” and the follow-up paper lack the population genetics perspective.*

2. The distributions of conserved repeat densities reported here, e.g. cTE/gTE seen in Figure 1, are uneven and lead the authors to conclude that there have been expansions and contractions of TE families that correspond to “three previously described periods regulatory innovations in vertebrate genomes.” I wonder if these apparent uneven distributions could be an artifact of the methods used whereby two extreme sets of repetitive sequences were used in the analysis: ancient repeats found in all mammals and human-specific repeats. What about families of repeats that are found in some, but not all, mammalian species including humans? Would inclusion of such families in the analysis change the distributions seen?

**Author’s response:***None of the 266 conserved repeats listed inAdditional file *2: *Table S2are human-specific. Eutrep families are present only in eutherian mammals and the cTE/gTE ratio for this group is ~ 0.17. The cTE/gTE ratio for platypus-specific families is ~0.3. Typically, the cTE/gTE ratio increases with the age of the family. The peaks in Figure*1*correspond to multiple families of similar age. Figure*1*and Figure*2*describe essentially the same uneven distributions obtained by two different approaches. We don’t see any room for artifacts.*

3. The majority of “conserved repeats” identified here are small fragments that cannot be related to any particular TE family. Do the authors feel that these are likely to be TE-derived as well? If they are not TE-derived how would that impact the conclusions relating the observations reported here to their previous work on the population dynamics of TEs? In other words, is it possible that many of regulatory innovations provided by the repetitive families observed here were not seeded via the fixation of TEs by drift in small populations, but rather by some other process?

**Author’s response:***The vast majority, if not all, of these fragments are likely to be TE-derived as well. After the paper was submitted, we classified over a dozen of conserved repeats as fragments of known TEs preserved in other vertebrates. The successful classification continues as new genomes become available (see also the response to the last reviewer). Even if some of the conserved repeats are not derived from TEs, they are unlikely to end up in our dataset, which was repeatedly filtered over the last five years, or so.*

4. Related to the point above. If the distributions showing clusters of conserved families in Figure 1 and Figure 2 of are broken down into those families of conserved repeats that can be demonstrated to be TE related versus the others, are similar uneven distributions seen? Do the TE-derived versus the non TE-derived distributions differ substantially and if so what are the implications?

**Author’s response:***We added Figure*1*A and Figure*1*B showing the distribution of the classified and unclassified repeats. The two peaks continue to be distinguishable. In Figure*1*A the first peak is larger than the second one. This simply illustrates that younger families of TEs are easier to classify than the older ones.*

Minor Points 5. The authors state that “Using binomial and chi square statistics we identified families composed of repetitive elements significantly overrepresented in the conserved regions relative to the rest of the genome.” and the analyses reported in the paper are based on those 266 conserved families. However, no data or information on this statistical analysis is provided in the Methods or Results sections. For instance, how significant are the overrepresentations seen? Do they vary widely? Are they different for TE-derived versus non TE-derived families (see points #3 and #4 above)?

**Author’s response:***Typically, older repeats are more significantly overrepresented in the conserved regions than the younger ones, due to continuing attrition of the corresponding non-conserved copies over time*. *We don’t see any other obvious patterns.*

6. Is it known whether populations of mammals were indeed more subdivided between periods of regulatory innovation as suggested by the authors on page 9-10?

**Author’s response:***The data suggest a more subdivided population during the evolutionary innovation periods and a less subdivided one when the periods end (i.e. between the periods). There is no direct way to determine the number of subpopulations that emerged and vanished during the evolutionary history of vertebrates. Based on our original hypothesis*[24]*, a surge in the number of subpopulations translates into an increase in the number of repetitive families in the genomic fossil record of a particular lineage. The surge is also likely to trigger a parallel surge in the number of new species and lineages, consistent with the punctuated equilibria theory. The analysis of the corresponding speciation patterns is possible based on the geological fossil record, but this goes beyond the scope of the paper.*

7. I did not see the Supplemental Materials and Methods cited by the authors in the descriptions of Figure 2 (only Supplemental Tables and Legends).

**Author’s response:***Corrected. The supplemental Materials and Methods are in the main text.*

Reviewer 3: JÃ¼rgen Brosius, University of Muenster, Germany

This manuscript describes detection of novel transposed elements in vertebrate mammalian and primate lineages and correlates their estimated times of expansion with regulatory innovations in vertebrate genomes. The authors detected ~150 additional "families of TEs" overrepresented in conserved regions of the genome. Some of these elements have similarities to known repeats, but most of them remained unidentified, thus far. The majority of newly described elements present in genomes are less than 150 copies. The "abundance" of some is less than 10 copies. It might be hard to discriminate between bona fide TEs and sequences that amplified via segmental duplications.

Here, the zinc-finger proteins or domains come to mind. This should be discussed and perhaps more information given as to why those elements are not derived from frequent segmental duplications or are merely retropseudogenes of, e.g., tRNAs or other small RNAs.

**Author’s response:***Based on our experience, the elements that are homologous to known pseudogenes, functional motifs and known TEs are relatively easy to identify. More difficult is to classify some of the LTRs and non-autonomous elements, which are often very diverse even in a single species. However, as indicated in Figures*1*A and*1*B, the observed patterns are quite similar for classified and unclassified families. As stated in the response to the second reviewer, the unclassified pile of conserved repeats continues to shrink as sequences from more vertebrate genomes become available.*

Perhaps the authors should spend more effort to bridge the gap between events that lead to the formation of sub-populations, which could happen over as little as a few hundred years or less and time frames of the proposed regulatory innovations during separation of the mammalian and bird lineages, the diversification of the mammalian lineages prior to the origin of eutherian lineages or even the diversification of mammals. Here, time spans of a few million up to tens of million years are probably involved. It is likely that very few, if any of the TEs insert are chock-full of pre-assembled, ready-to-use functional gene modules, such as enhancer sequences -- enhancers usually consisting of arrays of transcription factor binding sites that, with ~10 bp, are relatively short. In analogy to grape juice requiring further steps to generate wine, initially TEs are the raw material for innovation. As we have shown for the exaptation of (parts of) SINE elements as novel protein domains, it can take tens even 100 million years for such raw material to fortuitously acquire mutations that pave the way to functionality and exaptation [Krull M., Petrusma M., Makalowski W., Brosius J. & Schmitz J. (2007) Functional persistence of exonized mammalian-wide interspersed repeat elements (MIRs). Genome Res. 17:1139–45]. This is well beyond the time frames for formation of subpopulations.

**Author’s response:***We expanded on this point mostly in the section “Biological innovations and the diversity of repetitive families.“ The pattern of regulatory changes during a particular period of vertebrate evolution probably drove new biological adaptations. In fact, similar changes took place independently in different lineages*[14]*. Based on our original hypothesis*[24]*, we propose that the origin of new subpopulations was fueled by the new adaptations. Most subpopulations probably originated and became extinct in a relatively short time. The surviving subpopulations either diverged into separate species and lineages or channeled their TE-produced mutations back to the meta-population by crossbreeding. Therefore, the genomic DNA of the mammalian lineage combines mutations from countless subpopulations that existed at some point of time during the history of the lineage. These mutations likely contributed to the “raw material” fueling the genomic changes (see also the response to the first reviewer).*

In nature almost anything that is possible does happen and one cannot rule out such TE induced "regulatory hopeful monsters" resurrecting ideas of Richard Goldschmidt (see below) occasionally arise. However, one can imagine that a novel TE family with high copy number, that readily alters expression of most genes in whose vicinity it integrates, would cause havoc and would not bode well for the fitness of small populations.

**Authors' response:***This is quite correct. The extinction rate of small subpopulations is likely to be high and it may be an inherent part of the evolutionary process (see response to the first reviewer and “Repetitive families, population structure and speciation” in the main text).*

The idea that TEs contribute to speciation is not new and it is obvious that most of the time, the path leads via sub-populations. In the following, I am citing the work and ideas of other investigators from one of my review articles in [Brosius J: Disparity, adaptation, exaptation, bookkeeping, and contingency at the genome level. Paleobiology 2005, 31:S1-16]:

"Nevertheless, expression of the same gene at different times in development or in different cell types has long been suggested to be a key event in speciation

(Wilson 1975; Zuckerkandl 1975; Gould 1977b), and newly inserted retronuons are well capable of inducing such alterations […]. Without ignoring the potential of chromosomal rearrangements […] or even point mutations in a single gene, single retroposition events and, more likely, the combined impact of a newly arisen retronuon family (see also below) are reasonable scenarios that set the course for speciation (Bingham et al. 1982; Rose and Doolittle 1983; Ginzburg et al. 1984; McDonald 1990 […]). Apart from the significance of Hox genes and other developmental switches, I see the likely role of retroposition in speciation as a partial vindication of Richard B. Goldschmidt’s proposals concerning species-level saltations (Goldschmidt 1940; Gould 2002; Ronshaugen et al. 2002; Dietrich 2003; Wagner et al. 2003)."

Relevant references from the above quote:

Bingham, P. M., M. G. Kidwell, and G. M. Rubin. 1982. The molecular basis of P-M hybrid dysgenesis: the role of the P element, a P-strain-specific transposon family. Cell 29:995–1004.

Britten, R. J. 1996. DNA sequence insertion and evolutionary variation in gene regulation. Proceedings of the National Academy of Sciences USA 93:9374–9377.

Dietrich, M. R. 2003. Richard Goldschmidt: hopeful monsters and other ‘heresies.’ Nature Reviews Genetics 4:68–74.

Ginzburg, L. R., P. M. Bingham, and S. Yoo. 1984. On the theory of speciation induced by transposable elements. Genetics 107: 331–341.

Goldschmidt, R. B. 1940. Material basis of evolution. Yale University Press, New Haven, Conn.

Gould, S. J. 1977b. Ontogeny and phylogeny. Belknap Press of Harvard University Press, Cambridge.

Gould, S. J. 2002. The structure of evolutionary theory. Belknap Press of Harvard University Press, Cambridge.

McDonald, J. F. 1990. Macroevolution and retroviral elements. Bioscience 40:183–191.

Ronshaugen, M., N.McGinnis, andW.McGinnis. 2002.Hox protein mutation and macroevolution of the insect body plan. Nature 415:914–917.

Rose, M. R., and W. F. Doolittle. 1983. Molecular biological mechanisms of speciation. Science 220:157–162.

Wagner, G. P., C. Amemiya, and F. Ruddle. 2003. Hox cluster duplications and the opportunity for evolutionary novelties. Proceedings of the National Academy of Sciences USA 100: 14603–14606.

Wilson, D. S. 1975. A theory of group selection. Proceedings of the National Academy of Sciences USA 72:143–146.

Zuckerkandl, E. 1975. The appearance of new structures and functions in proteins during evolution. Journal of Molecular Evolution 7:1–57.

**Authors' response:***Indeed TEs likely contributed to the process due to their mutagenic activities and the role of small populations in speciation has been explored for quite some time. Our goal was to strictly focus on the origin of multiple repetitive families based on our original hypothesis*[24]*, and on the results presented in this paper. Nevertheless, both the origin of repetitive families and the origin of species are conceptually linked to small sub-populations. Therefore, in response to the thoughtful remarks presented in this and the previous review, we elaborated the text accordingly (see “Repetitive families, population structure and speciation”).*

Other points:

While clear to members of the scientific community working on transposable elements, the way transposable elements are described in the background sections of abstract and main body of the paper might give rise to some confusion and misunderstandings when addressing a broader audience. For example, it is not typical that transposable elements generate multiple interactive copies of themselves. In contrast, only a very small number (as little as one) master, source, or founder genes from which the proposed RNA template is transcribed are indeed multiplying THEMSELVES. Most integrated copies are not transcribed, available RNA copies being a prerequisite of retroposition. Hence, most TEs (with the exception of DNA transposons) are transposed elements with a small minority being transposABLE. This should be addressed throughout the text.

**Authors' response:***We revised the text as suggested.*

In the background section and throughout the manuscript, it should be clarified that TE families and the members decline via loss (elimination is for my taste too active a process) of DNA through recombination AND mutation of changes that, over time periods long enough, render such TEs virtually undetectable. TEs are mostly lost due to attrition. For example, the sentence on page 7 would be more precise as follows: "The ratio reflects a faster attrition of non-essential DNA in mouse than in human, driven by differences in the mutation rates between the two [16].

**Authors' response:***Attrition is an excellent term. We appreciate the suggestion and made the changes.*

Not only after fixation can TE derived repeats be recruited as functional components of the non-coding genomic DNA, but 1) can be exapted prior to fixation and 2) can also contribute to protein coding DNA as novel exons.

**Authors' response:***Any exaptation prior to fixation and other specific events are difficult to evaluate statistically. Therefore, our focus is mostly on the major evolutionary mechanisms leading to the observed rise and fall in the number of diverse families of TEs.*

I presume the characteristic hallmarks of DNA transposon's such as terminal inverted repeats are, after long time periods, only useful if they are long enough. The shorter terminal inverted repeats, like the short direct repeats of retroposons, will not be recognizable. See for example page 6 bottom, where the authors should explain which TE-specific features were applied.

**Authors' response:***Indeed, classification of ancient DNA transposons with short terminal inverted repeats (TIRs) is notoriously difficult. However, in most cases the classification is based on homology to known TEs, not on the presence or absence of putative TIRs. This is reflected in the annotations of individual conserved repeats.*

## Supplementary Material

Additional file 1 **Table S1.** Repbase entry names of TE families used in this study: (A) families from human and ancestral primates; (B) families from mouse and ancestral rodents; (C) families shared by all eutherian mammals and referred to as mammalian-wide families. (D) 266 families overrepresented in conserved regions of the human genome divided into: (E) 114 previously published families, (F) 152 families reported in this paper, (G) 25 families present in eutherian mammals only, (H) 130 families shared by mammals and chicken, and (I) 136 out of the 266 that are not present in chicken. Click here for file

Additional file 2 **Table S2.** Families of TEs overrepresented in conserved regions of the human genome. (A) family name; (B) repeat count in the conserved fraction of the human genome; (C) the total number of repeats in the human genome; (D) expected number of repeats in the conserved regions representing ~4.79% of the human genome; (E) expected number of repeats in the remaining portion of the human genome (95.21%); (F) binomial probability; (G) chi square. Columns H-N correspond to columns A-G, except that the numbers in columns B & C are based on repeat screening by Censor while the numbers in columns I & J are based on repeat screening by RepeatMasker. The family is considered significantly overrepresented if the binomial probabilities for both sets of repeat counts derived using Censor and RepeatMasker (columns F & M) are <5%, and the chi square values (columns G & N) also correspond to P<0.05. Click here for file
